# Hyperbaric Oxygen Therapy Alleviates Memory and Motor Impairments Following Traumatic Brain Injury via the Modulation of Mitochondrial-Dysfunction-Induced Neuronal Apoptosis in Rats

**DOI:** 10.3390/antiox12122034

**Published:** 2023-11-23

**Authors:** Reem Sakas, Katya Dan, Doron Edelman, Saher Abu-Ata, Aviv Ben-Menashe, Yaseen Awad-Igbaria, Jean Francois-Soustiel, Eilam Palzur

**Affiliations:** 1 Azrieli Faculty of Medicine, Bar-Ilan University, Safed 1311502, Israel; reem_sakas@hotmail.com (R.S.); katyashster@gmail.com (K.D.); saher9644@gmail.com (S.A.-A.); avivg091@gmail.com (A.B.-M.); jeans@gmc.gov.il (J.F.-S.); 2 Research Institute of Galilee Medical Center, Nahariya 221001, Israel; 3 Neurosurgery Department, Tel-Aviv Sourasky Medical Center, Tel-Aviv 6423906, Israel; dorone@tlvmc.gov.il; 4 Neurosurgery Department, Galilee Medical Center, Nahariya 221001, Israel

**Keywords:** traumatic brain injury, secondary brain injury, hyperbaric oxygen therapy (HBOT), apoptosis, mitochondria respiration

## Abstract

Traumatic brain injury (TBI) is a leading cause of morbidity and mortality in young adults, characterized by primary and secondary injury. Primary injury is the immediate mechanical damage, while secondary injury results from delayed neuronal death, often linked to mitochondrial damage accumulation. Hyperbaric oxygen therapy (HBOT) has been proposed as a potential treatment for modulating secondary post-traumatic neuronal death. However, the specific molecular mechanism by which HBOT modulates secondary brain damage through mitochondrial protection remains unclear. Spatial learning, reference memory, and motor performance were measured in rats before and after Controlled Cortical Impact (CCI) injury. The HBOT (2.5 ATA) was performed 4 h following the CCI and twice daily (12 h intervals) for four consecutive days. Mitochondrial functions were assessed via high-resolution respirometry on day 5 following CCI. Moreover, IHC was performed at the end of the experiment to evaluate cortical apoptosis, neuronal survival, and glial activation. The current result indicates that HBOT exhibits a multi-level neuroprotective effect. Thus, we found that HBOT prevents cortical neuronal loss, reduces the apoptosis marker (cleaved-Caspase3), and modulates glial cell proliferation. Furthermore, HBO treatment prevents the reduction in mitochondrial respiration, including non-phosphorylation state, oxidative phosphorylation, and electron transfer capacity. Additionally, a superior motor and spatial learning performance level was observed in the CCI group treated with HBO compared to the CCI group. In conclusion, our findings demonstrate that HBOT during the critical period following the TBI improves cognitive and motor damage via regulating glial proliferation apoptosis and protecting mitochondrial function, consequently preventing cortex neuronal loss.

## 1. Introduction

Traumatic brain injury (TBI) remains a leading cause of death and severe injuries worldwide across all ages [1]. As a result of TBI, damaged neurons are directly aimed at by a vast scale of complex and interlinked events that ultimately damage viable cells [2,3,4]. The significance of this phenomenon was addressed long ago by Reilly et al., who observed that many patients with head injuries remain conscious after the TBI but later die due to their injuries [5]. Suggesting that the primary injury may not be the main reason for a fatal outcome and drawing attention to secondary brain damage, setting in motion a vast and multidisciplinary research effort for the achievement of a better understanding of the underlying mechanisms and the development of novel therapeutic strategies for modulating the secondary brain damage [4,6].

Among the damaging events triggered by the injury, cerebral edema is widely recognized as one of the most significant threats in the progression of TBI [7,8], leading to swelling of the brain within the rigid skull, resulting in raised intracranial pressure (ICP) followed by impairment of cerebral perfusion and metabolism [9,10,11]. Therefore, ICP control has been the primary method of managing neuro-trauma, either based on hyperventilation, hypertonic solutions, or decompressive craniectomy. However, previous evidence suggests that oxidative metabolism collapse is not always ischemic in origin [12,13] and may even lead to ICP elevation [14]. Recent studies indicate that cerebral metabolism failure in brain injury is not necessarily mediated by ischemia and probably arises from mitochondrial damage and disruption of the electron transport chain [12,13].

The breakdown of the Ion gradients following TBI is regarded as a pivotal event that triggers a cascade of events, leading to cytotoxic edema, neuroinflammation, and neuronal swelling, ultimately leading to neuronal apoptosis [15]. Notable, the uncontrolled influx of Na^+^, Ca^2+^, and K^+^ plays a key role in the onset of cortical spreading depression (CSD) [16,17]. CSD is associated with multiple conditions, including TBI, migraine, and epileptic seizures [16,18,19], and it is characterized by the abruptly developing, near-complete, and sustained breakdown of transmembrane ion gradients, neurotransmitter release, increased energy metabolism, water shifts, and reduction of electric activity [20]. Prominently, the long-term depolarization could trigger intracellular signals and proteases, leading to neuronal death [20].

As a physiological protective mechanism, intracellular organelles such as mitochondria, endoplasmic reticulum, and Golgi absorb the excess ions [21,22,23]. However, an excessive accumulation of ions, such as Ca^2+^ within the mitochondrial matrix, can trigger the opening of the mitochondrial permeability transition pore (mPTP), resulting in the loss of mitochondrial membrane integrity and dissipation of the mitochondrial transmembrane potential (∆_Ψ_m), which reflects the loss of the proton gradient essential for the activation of ATP (Adenosine triphosphate) synthase [24]. Consequently, the failure of oxidative metabolism due to mitochondrial damage is probably the cause of spreading depression, cytotoxic edema, and neuronal swelling, all of which are involved in secondary injury [20,22].

It is conceivable that restoring mitochondrial function can contribute to restoring ATP cellular levels and subsequent control of cytotoxic edema through the re-activation of cation pumps and restoration of cellular homeostasis capability [25]. Hyperbaric oxygen therapy (HBOT) includes the inhalation of 100% oxygen at pressures exceeding 1-atmosphere absolute (ATA) to enhance the amount of oxygen dissolved in tissues [26]. Clinical studies have demonstrated that elevated levels of dissolved oxygen by HBOT can have several reparative effects on damaged brain tissues [27,28,29,30]. In the mean of secondary brain injury mechanisms, HBOT can promote vascular repair mechanisms, improve cerebral vascular flow [31,32,33], improve blood–brain barrier integrity, and reduce inflammatory reaction and edema [32,33,34,35,36].

Interestingly, HBOT can modulate the mPTP opening, thus has the potential to reverse this abnormality [37]. Previous animal models have reported improvements in mitochondrial function following HBOT [31] that were damaged after brain injury. The mitochondrial potential was fully restored to pre-injury value [38], and cellular ATP levels increased significantly [31]; hence, mitochondria membranes were preserved, and the mitochondrial pathway of apoptosis was reduced [31]. HBOT reduces the intrinsic mitochondrial pathway of apoptosis by reducing cytoplasm cytochrome c level, decreasing caspase enzyme’s activity, and modulation in Bax and Bcl-2 apoptotic proteins expression [30,39]. Although the neuroprotection of HBOT has been established in experimental animal models, it remains controversial in the clinic. Therefore, the current study aimed to examine hyperbaric therapy’s influence on various aspects, including motor and cognitive function, brain metabolism, pathological evaluation, and mitochondrial respiration function.

## 2. Materials and Methods

### 2.1. Animals

Sprague Dawley rats (Sex: Male; Age: 10 weeks; Weight: 250–300 g) obtained from Envigo RMS, Inc. (Rehovot, Israel) were used in the current study. All animal procedures were authorized by the Animal Care Committee of Bar-Ilan University (Approval #27-05- May 2016) and were carried out in accordance with the NIH Guide for the Care and Use of Laboratory Animals [40]. The animals were housed in groups of 3–4 in sterilized, solid-bottom cages with contact bedding under controlled temperature and a 12:12 h light/dark cycle. A standard pellet diet and water were provided ad libitum while all possible measures were taken to minimize the suffering of the animals.

### 2.2. Study Design

Spatial learning/reference memory and motor performance were measured before the Controlled cortical impact (CCI). Following the CCI, the animals were divided randomly into two groups: (1) The CCI without treatment (N = 14). (2) The CCI group treated with HBO (N = 14). The HBO treatment was performed after 4 h of the CCI and twice a day to day 4 after the CCI. Spatial learning/memory and motor performance were measured again between day 5 and day 13 after the CCI. At the end of the experiment, rats were sacrificed for histological analysis. In another experiment, following the CCI, the animals were randomly divided into three groups: (1) Naïve group without CCI (N = 6). (2) CCI without treatment (N = 6). (3) CCI group that was treated with HBO (N = 14). The HBO treatment was performed after 4 h of the CCI and twice a day to day 4 after the CCI. On day 5 following the CCI, rats were sacrificed for mitochondrial function measurement.

### 2.3. Controlled Cortical Impact (CCI) Model

TBI was produced using a modified technique previously described by Dean et al. [41]. Briefly, animals were anesthetized using 4% isoflurane within an induction chamber. Then, the animals were transferred and fixed in a stereotaxic rat frame (Stoelting, Wood Dale, IL, USA) while maintained under anesthesia through a nose cone with 2–3% isoflurane. Next, a longitudinal incision was made down the midline of the head to expose the skull. Following skull exposure, a 6 mm diameter craniotomy was made on the right hemisphere, 0.5 mm lateral to the sagittal suture and midway between the lambda and bregma sutures. Care was taken to avoid any dura damage.

Nevertheless, animals with damaged dura were excluded from the study. The exposed brain site was impacted with the Impact One Stereotaxic Impactor for CCI (Leica Biosystems, Wetziar, Germany), using a 5 mm diameter tip at a velocity of 5 m/s and a dwell time of 100 msec at a depth of 1 mm. Anesthesia was turned off briefly for a few seconds, allowing a breath of pure oxygen before the injury as a preventive measure against possible post-traumatic apnea. Following the impact, the craniotomy hole was blocked with bone wax, the scalp wound was sutured, and the animal was allowed to recover from anesthesia in an individual cage.

### 2.4. Hyperbaric Oxygen Therapy (HBOT)

HBOT intervention was executed per our previous works [30,42] in a hyperbaric research chamber for small animals (hipertech Inc., Istanbul, Turkey). After placing the CCI-HBOT group in the chamber, the chamber was flushed with 100% oxygen until it was full, with oxygen monitored using Servomex Oxygen Analyser 570A (Servomex group, Egham, UK). Then, the chamber was compressed to 2.5 Atmospheres Absolute (ATA) at a rate of 0.1 ATA per minute. Upon arrival at the designated depth time, counting of the treatment was started for 90 min.

### 2.5. Behavioral Tests

#### 2.5.1. Morris Water Maze

Morris water maze (MWM) was used to measure a new spatial reference memory task 1–5 days before and 1–5 days after brain injury [43]. The MWM consisted of a black circular pool 1.5 m in diameter, 40 cm deep, filled with warm water 26 ± 2 °C. A clear plexiglass platform 30.5 cm tall was submerged in the middle of one quadrant of the pool. The swim path and latency to find the hidden escape platform were monitored using a computer-controlled tracking system (EthoVisionXT, v15). Spatial cues were placed within each region (north, south, east, and west). The animals completed four trials, starting randomly from one of the pool’s four regions. However, if rats did not find the hidden platform in 120 s, they were placed directly on it for 30 s. Following each trial, rats were placed in a warmed cage for 4 min. The latency to the platform, average velocity, and time spent in the target quadrant with the platform were recorded. Animals were tested for 5 consecutive days before CCI as a baseline measure, and the same protocol was used on Days 5–9 after CCI to measure the outcome.

#### 2.5.2. Rotarod Test

Motor coordination and balance were assessed using the Rotarod test (Rat Rotarod NG, Model 47750; Ugo Basile, Varese, Italy) [44]. The apparatus consists of a rotating rod (6 cm diameter) with machined grips divided into four equal 8.7 cm wide sections raised 30 cm above trip boxes. Animals were trained to use the Rotarod one week before the CCI. In the training trials, animals were placed on the rod, which rotated at a constant speed of 10 rpm. The training trial continued until the animal could stay on the rod for 60 consecutive seconds without falling, turning around, or clinging to the rod. If they fell from the rod or turned around, they were correctly placed back on the rod, and the timer restarted. An accelerating protocol was used in test trials, where the rotation speed increased from 10 to 40 rpm over 300 s. Each trial was terminated if an animal fell, clung, rotated for two complete rotations, or remained on for more than 300 s. Latency to fall (s) was automatically recorded for each trial. The average of the three trials was calculated and used for analysis. The Rotarod apparatus was wiped with 30% ethanol and allowed to dry completely between animals.

### 2.6. Histology and Microscopy

#### 2.6.1. Tissue Collection

Animals were anesthetized, sacrificed, and transcardially perfused using heparinized saline solution, 10% sucrose in buffered saline, and 4% buffered formaldehyde. The brain specimens were excised and immersed in a 5% paraformaldehyde solution in phosphate buffer for an hour, after which they were embedded in paraffin. Subsequently, 5 μm thick sections were obtained from the coronal sections utilizing a microtome. The sections were then carefully mounted onto Matsunami adhesive glass slides (TM-1190 TOMO, MATSUNAMI, Kamogawa, Japan).

#### 2.6.2. Immunohistochemistry

Immunohistochemistry staining was performed as described before [45]. Briefly, the brain specimens’ slides were incubated for one hour at 60 °C before staining. Then, slides were deparaffinized using Xylene, Ethanol 100%, Ethanol 95%, Ethanol 70%, Ethanol 50% and DDW. Then, slides were subjected to heat-induced epitope retrieval using OmniPrep [pH 9.0, Cat. No. ZUC067-100, Zytomed Systems, Berlin, Germany] according to the manufacturer’s instructions. Briefly, the Coplin jar containing the slides in pre-warmed solution was placed in a water bath set to 85 °C for 20 min. Followed by 3× wash with DDW at 85 °C and rinsed with wash buffer (Zytomed systems, Berlin, Germany). Afterward, to remove background staining and eliminate non-specific binding, slides were incubated for 30 min with Background Buster (#NB306-50, INNOVEX, Saint-Nicolas, QC, Canada), followed by three cycles of wash buffer for two minutes each, then incubated for one hour at room temperature in a humidity chamber with one of the first antibodies: Rabbit anti-Caspase-3 (cleaved) (1:50, Cat. #RBK009-05, Zytomed systems, Berlin, Germany), Monoclonal-Anti-NeuN (1:50, Cat. # MAB377, Millipore corporations, Temecula, CA, USA) and Anti-Glial Fibrillary Acidic Protein (1:50, Cat. # M 0761, DakoCytomatio, Næstved, Denmark), then slides were incubated with sufficient peroxidase-labeled polymer-secondary antibody for 30 min, DAP for 2–5 min, and hematoxylin for 3 min, after each of these steps, the slides were thoroughly rinsed three times with a wash buffer for one minute each, then slides were sequentially into 60%, 80%, 90%, 100% ethanol, and Xylene bath for 5 min each. Afterward, a glass cover was affixed on the slides and sealed with glue.

### 2.7. Microscopy

Microscopic observation was performed using the Eclipse Ci microscope (Nikon Corp., Kamogawa, Japan). Quantitative assessment was performed in two different noncontiguous sections randomly cut through the lesion, both in the perilesional area and the corresponding area in the opposite hemisphere. Randomly chosen digital microphotographs of both slices were obtained at 200× magnification. At least 10 visual fields in each slice were acquired by the Nikon DS-Ri1 camera (Nikon Corp., Kamogawa, Japan) with the same microscope settings and exposure time for averaging the number of positively stained cells for each hemisphere. The images were analyzed using the ImageJ software 1.46 (NIH Image J, National Institutes of Health, Bethesda, MD, USA). The number of positively stained cells determined the number of Cas3, Neun, and GFAP. The images were analyzed by an observer blinded to animal grouping.

### 2.8. Mitochondrial Function

#### 2.8.1. Mitochondria Isolation from Rat Brain

Mitochondria from rat brains were isolated according to the manufacturer’s instructions for the mitochondria isolation kit (Mito-Iso1, Sigma-Aldrich, Saint Louis, MO, USA). Briefly, tissue samples were harvested from the injured region of the cerebral hemisphere. The tissue samples were washed with MiR05 (110 mM sucrose, 60 mM K^+^-lactobionate, 0.5 mM EGTA, 3 mM MgCl_2_, 20 mM taurine, 10 mM KH_2_ PO_4_, 20 mM HEPES adjusted to pH 7.1 with KOH, and 1 g/L BSA essentially fatty acid-free). Then, the brain tissue (200–250 mg) was homogenized with 10 volumes of extraction buffer (5 mM EGTA, 1 M D-mannitol, 350 mM Sucrose, 50 mM HEPES adjusted to pH 7.5 with NaOH, and 1 g/L BSA essentially fatty acid-free) using a pestle and glass tube. The homogenate was centrifuged at 600× *g* for 10 min. The supernatant was centrifuged at 11,000× *g* for 10 min. After this, the supernatant was removed, and the pellet was resuspended in 10 vol. of extraction buffer (5 mM EGTA, 1 M D-mannitol, 350 mM Sucrose, 50 mM HEPES adjusted to pH 7.5 with NaOH) and centrifuged at 600× *g* for 10 min. Finally, the supernatant was centrifuged at 11,000× *g* for 10 min, and the isolated mitochondria were resuspended in MiR05. The concentration of mitochondrial proteins was assessed using the Bradford assay, employing the Pierce Coomassie Plus Protein Assay. The analysis utilized bovine serum albumin (BSA) as a standard.

#### 2.8.2. Mitochondrial Respiration Measurements

High-resolution respirometry was performed on freshly acquired brain tissue samples utilizing the high-resolution respirometer OROBOROS Oxygraph-2k apparatus (Oroboros Instruments, Innsbruck, Austria). According to the manufacturer’s guidelines, the oxygraph was calibrated at 37 °C, and each chamber was filled with 2 mL of MiR05 and allowed to stabilize for 40 min. To investigate various mitochondrial coupling control states and electron transfer pathways, we employed the SUIT-020 Fluo mt D033 protocol [46], enabling the precise assessment of O_2_ flux in isolated mitochondria. This comprehensive protocol facilitated the evaluation of mitochondrial function under diverse conditions, including basal respiration (measuring oxygen consumption without any intervention), non-phosphorylating LEAK respiration, representing uncoupled oxygen consumption unrelated to ATP synthesis, OXPHOS state reflecting oxygen consumption coupled to ADP phosphorylation for maximal ATP production, and ET capacity, measuring oxygen consumption when mitochondria are uncoupled from ATP synthesis using an optimal concentration of an uncoupler. Notably, specific steps were as follows: brain mitochondrial protein concentration of 0.25 mg/mL, induction of LEAK state with pyruvate (5 mmol/L) and malate (5 mmol/L), the establishment of OXPHOS state for the NADH pathway through complex I using ADP (2.5 mmol/L) and glutamate (2.5 mmol/L), assessment of combined NADH and succinate pathways (NS pathway) with succinate (10 mmol/L), evaluation of succinate pathway through complex II by adding rotenone (1 μmol/L), induction of Leak-resting State by inhibiting ATP synthase with oligomycin (2.5 mmol/L), determination of electron transfer (ET) capacity using CCCP (2.5 mmol/L), and measurement of non-mitochondrial residual oxygen consumption (ROX) after inhibiting complex III with antimycin A (5 μmol/L). Mitochondrial respiration quantification was expressed as flux per mass, denoted as pmol O_2_/s/mg (picomoles of oxygen consumed per second per milligram of wet tissue mass).

### 2.9. Data Analysis

Data analysis was conducted using IBM SPSS statistics version 26 and GraphPad Prism9, with all results presented as the Mean ± SEM. Spatial learning/reference memory and locomotor performance between sessions were assessed using mixed model repeated measures analysis of variance (ANOVA). Significant main effects and interactions were further pursued using an independent sample *t*-test. Differences between groups were assessed using a student *t*-test, or one-way ANOVA, followed by Post hoc Tukey’s test. The accepted significance value for all tests was set at *p* < 0.05.

## 3. Results

### 3.1. HBOT Controls the Development of Spatial Learning Impairment after CCI

**Figure 1:**
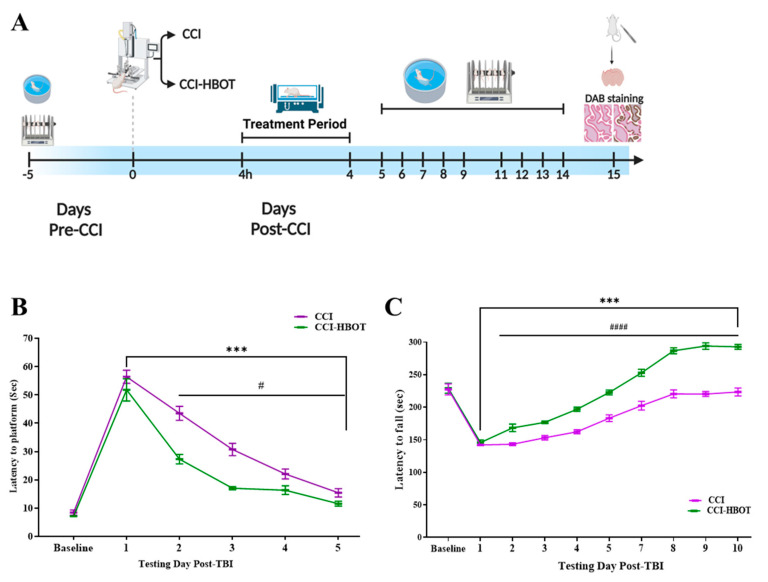
The early HBO treatment modulates memory and motor dysfunction following CCI. (**A**) The experimental timeline. Spatial memory and motor performance were measured before and after the CCI. After CCI, animals were randomly assigned to one of the experiment groups: CCI group. CCI-HBOT group. The HBO treatment was performed after 4 h of the CCI and twice a day to day 4 after the CCI. The animals were sacrificed at the end of the study. (**B**) The Morris water maze testing established similar learning curves in the two groups before the injury. Following CCI, treatment with HBO led to a significant improvement in the learning curve, expressed by a reduced latency to reach the platform. (**C**) The sensorimotor recovery following CCI and CCI-HBOT were tested using the rotarod test. Motor performance is expressed by latency to fall (Second). Treatment with HBO significantly improved motor performance compared to the CCI group. Mixed Model ANOVA; Student *t*-test; Paired sample *t*-test. Mean ± SEM. *** *p* < 0.001 Compared to the 1ST test session following the CCI; ^#^ *p* < 0.05, ^####^ *p* < 0.001 Difference between groups.

Before the CCI was applied, a repeated measures GLM analysis was conducted to compare the performance of the CCI and CCI-HBOT groups across five time points. The analysis revealed significant main effects of Time, indicating a decrease in latency time to reach the platform [F_(4,104)_ = 98.59, *p* < 0.001, η^2^ = 0.791]. However, there was no significant main effect of Group [F_(1,26)_ = 1.054, *p* = 0.314, η^2^ = 0.039], and no significant Group × Time-points interaction [F_(4,104)_ = 0.302, *p* = 0.864, η^2^ = 0.012]. These findings suggest no differences in the performance levels or improvement rates between the two groups before the CCI (Figure 1B).

Following the CCI, there was a significant main effect of Time on the latency to reach the platform [F_(4,104)_ = 174.078, *p* < 0.001, η^2^ = 0.87, Figure 1B]. Additionally, there was a significant main effect of Group on the latency to reach the platform [F_(1,26)_ = 19.792, *p* < 0.001, η^2^ = 0.432, Figure 1B], along with a significant Group × Time-points interaction [F_(4,104)_ = 5.459, *p* < 0.001, η^2^ = 0.174, Figure 1B]. These results indicate different performance levels and a difference in the rate of improvement between the CCI and the CCI-HBOT group over the five days of the experiment (Figure 1B). The interaction was driven by a superior performance of the CCI-HBOT group in the latency to reach the platform on each day-test (Excluding the first day of the test after TBI) compared to the CCI group (Day1 t_(26)_ = −1.011, *p* = 0.32; Day2 t_(26)_ = −5.40, *p* < 0.001; Day3 t_(26)_ = −6.07, *p* < 0.001; Day4 t_(26)_ = −2.45, *p* = 0.021; Day5 t_(26)_ = −2.22, *p* = 0.035, Figure 1B).

### 3.2. HBOT Regulates the Progression of Motor Impairment Following CCI

Before CCI, there was no significant difference in motor performance (Latency to fall) between the CCI group and CCI-HBOT group (t_(26)_ = 0.164, *p* = 0.8, Figure 1C). Thus, both groups show similar motor performance in the rotarod test (Figure 1C). Following CCI, both groups show a significant reduction in latency to fall compared to the baseline (paired sample *t*-test, t_(13)_ = 10.924, *p* < 0.001, t_(13)_ = 10.169, *p* < 0.001, CCI-HBOT and CCI group, respectively, Figure 1C). With no significant difference between the groups (t_(26)_ = 0.814, *p* = 0.42, Figure 1C).

To further investigate performance differences between the CCI-HBOT and CCI groups across time points, a repeated measures GLM analysis was conducted, considering Time as the main factor within subjects and Group as the between-subject factor. The analysis revealed significant main effects of Time, indicating an increased latency time to fall [F_(8,208)_ = 207.6, *p* < 0.001, η^2^ = 0.889, Figure 1C]. Moreover, there was a significant main effect of Group on performance [F_(1,26)_ = 494.5, *p* < 0.001, η^2^ = 0.95], and a significant interaction between Group × Time-points interaction [F_(8,208)_ = 14.06, *p* < 0.001, η^2^ = 0.351]. These findings indicated that the performance levels and the rate of improvement varied between the two groups throughout the 9-test sessions (Figure 1C).

The interaction was driven by a superior performance of the CCI-HBOT group in the latency to fall on each day-test (Excluding the first day of the test after CCI), compared to the CCI group (Day1 t_(26)_ = 0.81, *p* = 0.42; Day2 t_(26)_ = 4.06, *p* < 0.001; Day3 t_(26)_ = 6.05, *p* < 0.001; Day4 t_(26)_ = 7.53, *p* < 0.001; Day5 t_(26)_ = 6.19, *p* < 0.001; Day7 t_(26)_ = 5.87, *p* < 0.001; Day8 t_(26)_ = 8.75, *p* < 0.001; Day9 t_(26)_ = 11.65, *p* < 0.001, Day10 t_(26)_ = 9.72, *p* < 0.001, Figure 1C). Notable, both groups showed an increase in latency to fall between sessions [F_(8,104)_ = 168.289, *p* < 0.001, η^2^ = 0.928; F_(8,104)_ = 56.394, *p* < 0.001, η^2^ = 0.813; CCI-HBOT group, CCI group, respectively, Figure 1C].

### 3.3. HBOT Prevents Neuronal Loss and Glial Cell Proliferation Following CCI

**Figure 2:**
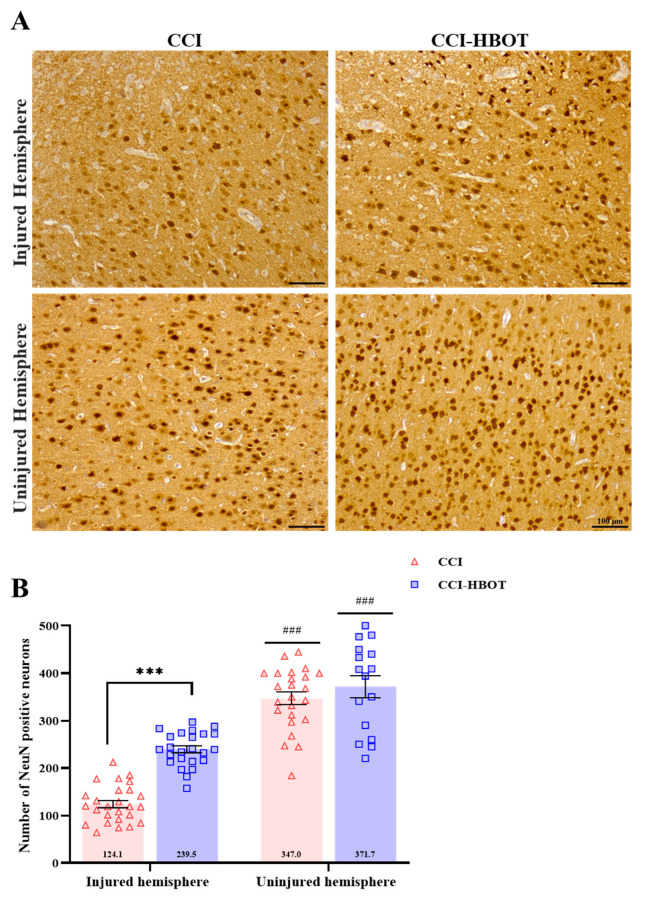
HBOT prevents cortical neuronal loss following CCI. (**A**) Cortical neuronal staining. Representative images of brain neuronal staining with anti-NeuN in CCI and CCI-HBOT in the injured (**upper panel**) and uninjured hemisphere (**lower panel**). Scale bar: 100 µm. (**B**) The number of positive NeuN-labeled cells. NeuN expression was significantly reduced in the injured hemisphere compared with the uninjured hemisphere in both groups. A significantly lower expression of NeuN (injured hemisphere) was noted in the CCI group compared to the CCI-HBOT group. Mean ± SEM. One-way Anova followed with Tukey’s test. *** *p* < 0.0001 Difference between groups; ^###^ *p* < 0.0001 Difference between injured and uninjured hemispheres of the same group.

Following CCI, a reduction in the number of cortical neurons was observed in the injured hemisphere of the CCI and the CCI-HBOT group compared to the uninjured hemisphere [F_(3,86)_ = 83.57, *p* < 0.001, Figure 2A,B). In the injured hemisphere, the HBOT demonstrated significant prevention of neuronal loss, resulting in significantly higher numbers of neurons in the CCI-HBOT groups compared to the CCI group (*p* < 0.001, Figure 2A,B).

**Figure 3:**
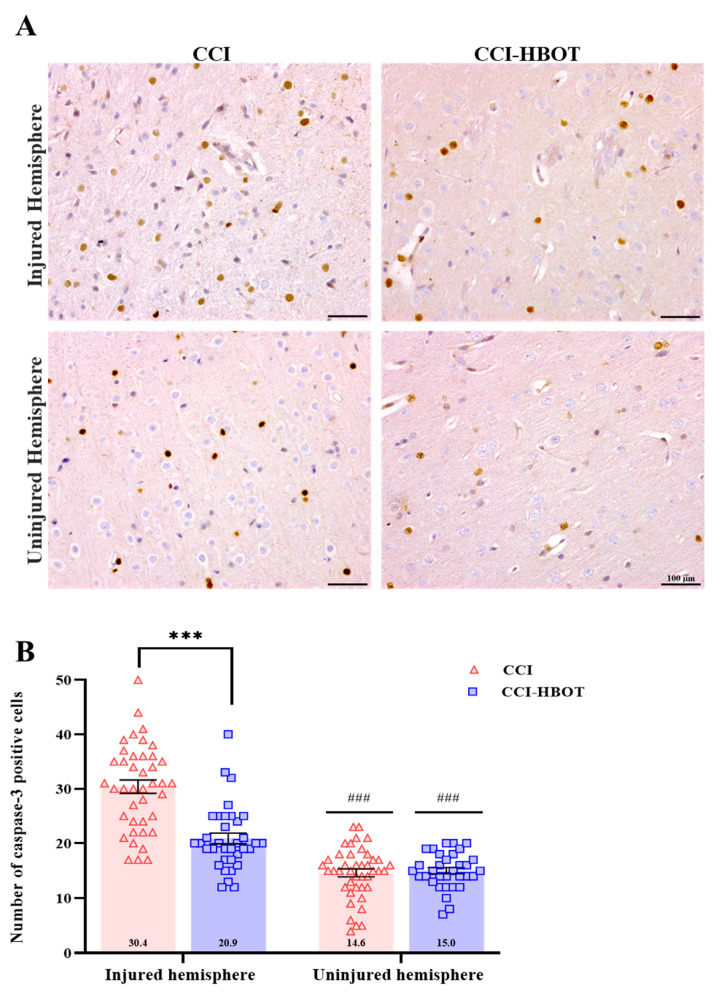
CCI induces a robust level of cortical apoptosis. (**A**) Cortical activated caspase-3 staining. Representative images of cortical apoptosis staining with anti-Caspase3 in CCI and CCI-HBOT in the injured (**upper panel**) and uninjured hemisphere (**lower panel**). Scale bar: 100 µm. (**B**) Number of positive caspase-3 labeled cells. Caspase-3 expression was significantly increased in the injured hemisphere compared with the uninjured hemisphere in both groups. A significantly lower expression of caspase-3 (injured hemisphere) was noted in the CCI-HBOT group compared to the CCI group. Mean ± SEM. One-way Anova followed with Tukey’s test. *** *p* < 0.0001 Difference between groups; ^###^ *p* < 0.0001 Difference between injured and uninjured hemispheres of the same group.

In addition, apoptotic cell death, indicated by activated caspase3 expression, was evaluated in the context of CCI. We found a significant increase in the number of positively stained cells with caspase3 in the injured hemisphere of the CCI and the CCI-HBOT group compared to the uninjured hemisphere [F_(3,147)_ = 65.27, *p* < 0.001, Figure 3A,B]. In the injured hemisphere, remarkably, HBOT intervention led to a significant reduction in apoptotic cell count (*p* < 0.001, Figure 3A,B).

**Figure 4:**
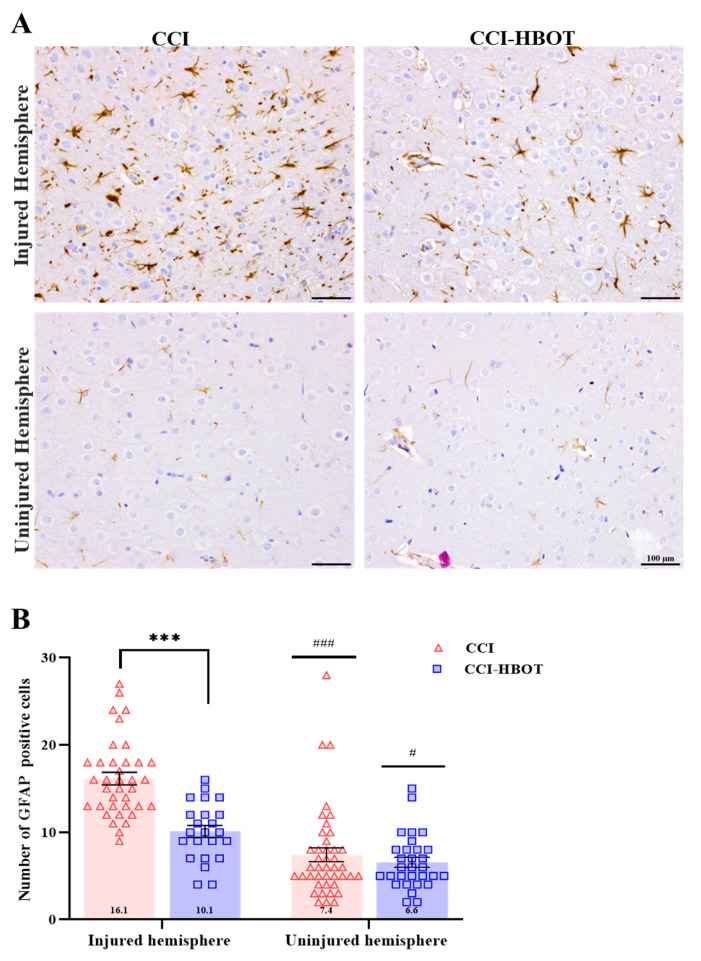
The early HBO treatment modulates cortical glial proliferation following CCI (**A**) Glial cell staining. Representative images of cortical GFAP staining in CCI and CCI-HBOT in the injured (**upper panel**) and uninjured hemisphere (**lower panel**). Scale bar: 100 µm. (**B**) Number of positive GFAP-labeled cells. GFAP expression was significantly increased in the injured hemisphere compared with the uninjured hemisphere in both groups. A significantly higher expression of GFAP (injured hemisphere) was noted in the CCI group compared to the CCI-HBOT group. Mean ± SEM. One-way Anova followed with Tukey’s test. *** *p* < 0.0001 Difference between groups; ^#^ *p* < 0.05, ^###^ *p* < 0.0001 Difference between injured and uninjured hemispheres of the same group.

The number of glial cells was assessed to evaluate the presence of glial cells specifically marked by GFAP. An increase in glial cells was observed (Figure 4A), indicating gliosis in the injured hemisphere of the CCI and the CCI-HBOT group compared to the uninjured hemisphere [F_(3,128)_ = 37.15, *p* < 0.001, Figure 4A,B]. In the injured hemisphere, we found that HBO treatment significantly reduced the GFAP-stained glial cells compared to the untreated group (CCI, *p* < 0.001, Figure 4B).

### 3.4. HBOT Restores Mitochondrial Respiration Following CCI

**Figure 5:**
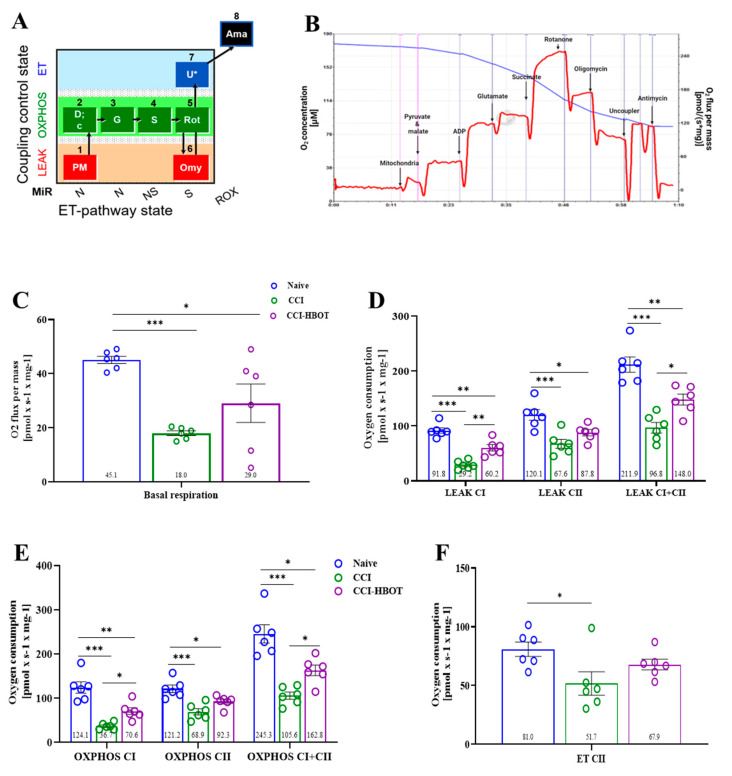
The effect of CCI on mitochondrial oxygen consumption. (**A**) Respiratory SUIT protocol. Different steps and respiratory states were tested by adding various substrates, uncouplers, and inhibitors. Providing a comprehensive overview of the experimental design and methods used to investigate mitochondrial function following CCI injury and HBOT treatment. (**B**) An illustration of the experiment is when oxygen consumption rates are measured in response to adding pyruvate–malate, glutamate, ADP, succinate rotenone, oligomycin, CCCP, and antimycin. Blue-Thin line represents the oxygen concentration of the chamber (left *y* axis) and the oxygen flux per mass (right *y* axis). (**C**) Basal mitochondrial respiration in the CCI and CCI group treated with HBO for 4 days. (**D**) Mitochondrial respiration in the non-phosphorylation resting states-LEAK. (**E**) Mitochondrial oxygen consumption in the OXPHOS states coupled to ADP phosphorylation for maximal ATP production. (**F**) Mitochondrial oxygen consumption in the ET capacity state. (N = 6) (Respiratory flux is expressed in pmol O_2_ per second per milligram wet tissue mass-pmol O_2_/s/mg). Mean ± SEM. One-way Anova followed with Tukey’s test. * *p* < 0.05, ** *p* < 0.001, *** *p* < 0.0001.

Mitochondrial oxygen consumption in rat brain mitochondria was investigated 4 days following CCI to assess mitochondrial function across various respiration states, including the leak state, oxidative phosphorylation state with NADH pathway activation (OXPHOS CI, Figure 5A), and oxidative phosphorylation state with succinate pathway activation (OXPHOS CII, Figure 5A).

The CCI and the CCI-HBOT group show a significant reduction in the mitochondrial oxygen consumption in the basal respiration state compared to the naïve group [F_(2,15)_ = 10.747, *p* < 0.001, Figure 5C]. Notably, we noted a slight improvement in the basal respiration of the CCI-HBOT group compared to the CCI group (Figure 5C). Moreover, in the leak state, we observed a significant reduction in mitochondrial oxygen consumption in the CCI and CCI-HBOT group compared to the naïve group [F_(2,15)_ = 43.608, *p* < 0.001; F_(2,15)_ = 10.954, *p* < 0.001; F_(2,15)_ = 25.339, *p* < 0.001, LEAK CI, LEAK CII, LEAK CI + CII, respectively, Figure 5D]. Remarkably, the early HBOT significantly improved mitochondrial oxygen consumption in the leak states compared to the CCI group (*p* < 0.05, Figure 5D).

In the CCI group, a significant reduction in mitochondrial oxygen consumption was observed in the maximal ATP production, together with the activation of CI and CII, compared to the naïve and CCI-HBOT group [F_(2,15)_ = 25.319, *p* < 0.001, F_(2,15)_ = 13.529, *p* < 0.001, F_(2,15)_ = 23.296, *p* < 0.001, OXPHOS CI, OXPHOS CII, OXPHOS CI&CII, respectively, Figure 5E]. Thus, the naive and CCI-HBOT group exhibited higher mitochondrial oxygen consumption in oxidative states compared to the CCI group (Figure 5E). In addition, in the electron transfer capacity state, we found that CCI reduces the mitochondrial ET capacity compared to the naïve group [F_(2,15)_ = 4.061, *p* = 0.039, Figure 5F]. Notable, no difference was observed between the CCI-HBOT group, CCI, and the naïve group (Figure 5F).

## 4. Discussion

TBI is frequently a complex and devastating condition that lacks a definitive cure. Despite years of research, the focus remains on finding effective therapeutic interventions to modulate the secondary brain damage that develops after the primary mechanical impact [42]. The current study highlights several cellular and molecular factors involved in secondary brain damage. Thus, we found that TBI led to spatial learning and motor dysfunction, which were associated with elevated levels of apoptosis, overactivation of glial cells, neuronal loss, and mitochondrial dysfunction in the injured hemisphere. Critically, however, we found that HBO treatment, which has been reported to exert anti-inflammatory and neuroprotective effects, significantly modulates the secondary brain damage, probably through reducing hypoxia and ROS production and increasing the oxygen content in circulation and tissue perfusion [42,47,48].

Numerous studies have demonstrated the neuroprotective effects of HBO treatment in various models of brain injury [30,42]. Thus, some evidence indicates that HBO therapy has beneficial effects in promoting tissue recovery and cellular metabolism and improving functional outcomes after TBI [42,49,50]. Notably, cognitive dysfunction is a common consequence of TBI, significantly impacting an individual’s quality of life [51]. Our findings revealed that HBOT improves spatial learning and reference-memory performance following CCI. Thus, the CCI-HBOT group significantly reduces the latency time to reach the platform in the MWM test. The current result aligns with previous findings that have shown a beneficial effect of HBOT on cognitive impairments linked to TBI [51,52,53].

Furthermore, motor impairment is frequently reported among TBI patients, and it affects the individual’s mobility and independence [54]. In the current study, we assessed the effect of HBO treatment on motor coordination using an accelerating rotarod test. While there were no significant differences in motor performance between the CCI group and CCI-HBOT group before and immediately after the injury, a notable divergence in performance was observed between the two groups over time. The CCI-HBOT group exhibited a more favorable rate of improvement in motor coordination than the CCI group. Thus, our results indicate that HBOT intervention can potentially improve motor function following CCI.

Interestingly, improved motor performance was observed in the CCI group. Such improvement may stem from the long-term procedural motor memory induced by repeated practice, a well-documented phenomenon in both humans and laboratory animals [55,56,57,58]. Therefore, a part of the motor performance enhancement in both groups (CCI, CCI-HBOT) is probably attributed to the mnemonic processes associated with task practice.

Neuronal damage and apoptosis are critical pathological events in TBI [59]. The primary mechanical impact and subsequent biochemical cascades can lead to widespread neuronal death and apoptosis, further contributing to brain damage [60]. Strikingly, our result indicates that HBO treatment significantly modulates neuronal death and reduces apoptosis in the injured hemisphere. Thus, the CCI-HBOT group displayed a higher number of surviving neurons and a significant decrease in apoptotic cell count compared to the CCI group. These findings highlight the neuroprotective effects of HBOT, suggesting its potential as a therapeutic tool to modulate neuronal damage and apoptosis in TBI.

Furthermore, HBO treatment has shown a beneficial effect in regulating the overactivation of glial cells following TBI. The glial cell plays a crucial role in the immune response and tissue repair following TBI [61,62]. Here, we found that HBO treatment can reduce the number of glial cells involved in the gliosis process, a generalized central nervous system reaction to tissue injury [63]. Gliosis involves the proliferation or hypertrophy of various types of glial cells, including astrocytes, microglia, and oligodendrocytes [63]. The decrease in glial cell count suggests that HBOT restricts secondary brain damage and regulates the gliosis process.

Neuroinflammation is a common factor in TBI [64]. Thus, over-release of pro-inflammatory cytokines and elevated microglia and glial activation levels are frequently reported following brain injury [60]. Neuroinflammation contributes to secondary brain damage by elevating neuronal death, edema, and oxidative stress [60,65]. It is conceivable that regulating the neuroinflammation process might be a potential prevention approach for modulating secondary brain damage. Therefore, we speculate that the beneficial effect of the HBO treatment may stem from its previously documented anti-inflammatory properties [66,67,68].

Our previous studies have shown that nerve injury in TBI and neuropathic pain conditions involves mitochondrial dysfunction [25,69]. Mitochondria are vital cellular organelles responsible for energy production, and their dysfunction can lead to impaired cellular metabolism and increased vulnerability to cell death [25,69]. Our results demonstrated that CCI impaired mitochondrial respiration in different challenging states. Thus, the CCI groups have shown reduced mitochondrial oxygen consumption in the basal, leak, OXPHOS CI, and OXPHOS CII states. However, the early HBO treatment significantly improved mitochondrial oxygen consumption in the oxidative phosphorylation state (i.e., phosphorylation of ADP to ATP) and leak respiration state compared to the CCI group. The diminished level of mitochondrial oxygen consumption probably results from a decrease in membrane potential (ΔΨm) that occurs under metabolically stressful conditions, such as neuroinflammation, that requires high ATP demand and activation of the mitochondrial protective mechanism to mitigate ROS [70,71].

The mitochondria consume approximately 90% of the incoming oxygen into the cell; therefore, elevating the oxygen levels through HBO administration might directly impact mitochondrial activity [72,73,74]. HBO can improve mitochondrial redox, preserve mitochondrial integrity, activate transcription factors, relieve oxidative stress, and promote neuroprotection effects following TBI [30,73]. Moreover, HBO treatment can enhance the ETC by amplifying its electron acceptor capacity. Hence, by elevating oxygen levels, an expanded oxygen reserve is available for electron acceptance [73,75]. Enhancing the electron acceptor capacity, in turn, probably alleviates the energy crisis characterized by ATP depletion that arises during metabolically challenging situations, such as neuroinflammation, CSD, and ion gradient disturbances.

The study of HBOT in TBI has yielded mixed results, and the effects of oxygen tension on mitochondrial function have been debated [76]. While some studies have suggested that high oxygen tension can increase oxidative stress in mitochondria [77], others have demonstrated that decreased oxidative stress induced by HBOT can have a protective effect, particularly in the context of ischemia-reperfusion injury [78]. These discrepancies may be attributed to variations in pressure settings, treatment duration, and the specific pathophysiological conditions of the injury. Further research is needed to elucidate the optimal pressure and duration of HBOT to achieve the desired therapeutic effects in TBI.

Despite the valuable insights gained from our study, it is essential to acknowledge its limitations. Further research is necessary to determine the optimal therapeutic protocol for HBOT in the context of TBI. Factors such as the timing and duration of HBOT and the pressure used may influence its efficacy and should be systematically explored. Additionally, long-term follow-up studies are needed to assess the sustained effects of HBOT on cognitive function, motor performance, and neuronal health in TBI models.

## 5. Conclusions

Our findings suggest that cognitive and motor dysfunction resulting from TBI is mediated by elevated levels of apoptosis, neuronal loss, overactivation of glial cells, and mitochondrial dysfunction. Crucially, HBO treatment during the secondary brain injury phase improves mitochondrial functions and regulates neuronal loss and glial proliferation, resulting in superior cognitive and motor performance in the HBOT group. Our findings highlight the therapeutic potential of HBOT in the management of TBI.

## Data Availability

Data is contained within the article. In addition, datasets examined in the present study can be obtained by contacting the corresponding author.

## Abbreviations

TBI	Traumatic brain injury
CCI	Controlled cortical impact
HBOT	Hyperbaric oxygen therapy
ICP	Intracranial pressure
ROS	Reactive oxygen species
TSPO	Translocator protein
CI	Complex I
CII	Complex II
ET	Electron Transfer
mPTP	Mitochondrial permeability transition pore
